# RACK1 is evolutionary conserved in satellite stem cell activation and adult skeletal muscle regeneration

**DOI:** 10.1038/s41420-022-01250-8

**Published:** 2022-11-18

**Authors:** Elisabetta Catalani, Silvia Zecchini, Matteo Giovarelli, Agnese Cherubini, Simona Del Quondam, Kashi Brunetti, Federica Silvestri, Paulina Roux-Biejat, Alessandra Napoli, Silvia Rosanna Casati, Marcello Ceci, Nicla Romano, Silvia Bongiorni, Giorgio Prantera, Emilio Clementi, Cristiana Perrotta, Clara De Palma, Davide Cervia

**Affiliations:** 1 Department for Innovation in Biological, Agro-food and Forest systems (DIBAF), Università degli Studi della Tuscia, largo dell’Università snc, 01100 Viterbo, Italy; 2 Department of Biomedical and Clinical Sciences “Luigi Sacco” (DIBIC), Università degli Studi di Milano, via G.B. Grassi 74, 20157 Milano, Italy; 3 Department of Medical Biotechnology and Translational Medicine (BioMeTra), Università degli Studi di Milano, via L. Vanvitelli 32, 20129 Milano, Italy; 4 Department of Ecological and Biological Sciences (DEB), Università degli Studi della Tuscia, largo dell’Università snc, 01100 Viterbo, Italy; 5 Unit of Clinical Pharmacology, University Hospital “Luigi Sacco”-ASST Fatebenefratelli Sacco, via G.B. Grassi 74, 20157 Milano, Italy; 6 Scientific Institute IRCCS “Eugenio Medea”, via Don Luigi Monza 20, 23842 Bosisio Parini (LC), Italy

**Keywords:** Cell biology, Physiology

## Abstract

Skeletal muscle growth and regeneration involves the activity of resident adult stem cells, namely satellite cells (SC). Despite numerous mechanisms have been described, different signals are emerging as relevant in SC homeostasis. Here we demonstrated that the Receptor for Activated C-Kinase 1 (RACK1) is important in SC function. RACK1 was expressed transiently in the skeletal muscle of post-natal mice, being abundant in the early phase of muscle growth and almost disappearing in adult mature fibers. The presence of RACK1 in interstitial SC was also detected. After acute injury in muscle of both mouse and the fruit fly *Drosophila melanogaster* (used as alternative in vivo model) we found that RACK1 accumulated in regenerating fibers while it declined with the progression of repair process. To note, RACK1 also localized in the active SC that populate recovering tissue. The dynamics of RACK1 levels in isolated adult SC of mice, i.e., progressively high during differentiation and low compared to proliferating conditions, and RACK1 silencing indicated that RACK1 promotes both the formation of myotubes and the accretion of nascent myotubes. In Drosophila with depleted RACK1 in all muscle cells or, specifically, in SC lineage we observed a delayed recovery of skeletal muscle after physical damage as well as the low presence of active SC in the wound area. Our results also suggest the coupling of RACK1 to muscle unfolded protein response during SC activation. Collectively, we provided the first evidence that transient levels of the evolutionarily conserved factor RACK1 are critical for adult SC activation and proper skeletal muscle regeneration, favoring the efficient progression of SC from a committed to a fully differentiated state.

## Introduction

Adult skeletal muscle retains the capability to increase in size upon hypertrophic stimuli and to regenerate after injury by a hyperplasic process. Although mature muscle cells possess the capacity to self-repair [1, 2], the main process underlying regeneration involves the activity of postnatal resident stem cells, namely satellite cells (SC) [3]. SC play a role also in response to increased muscle load, such as after exercise, and contribute to the efficient growth of adult myofibers. SC are maintained under quiescent conditions, histologically located in a niche environment between the sarcolemma and the basal lamina of the muscle fiber as small spindle-shaped elements. After stimulation, SC cells change their cellular structure, expanding the cytoplasmic portion, including organelles. In parallel, SC proliferate, differentiate and fuse either each other to form new myofibers or with pre-existing fibers to increase their size [3–6]. The new muscle fibers translate for all the sarcomere components. Activated SC can engage both symmetric and asymmetric divisions. Asymmetric division is a key mechanism that allows the maintenance of the stem cells pool by supporting the balance between self-renewal and differentiation [3–6]. Once regeneration is complete, SC re-enter quiescence and skeletal muscle regains homeostasis.

Cells of myogenic lineage originate from Pax3^+^/Pax7^+^ progenitors which are both downregulated during active myogenesis, when myogenic regulatory factors, such as MyoD and MyoG, are induced. The main cell-intrinsic molecular mechanisms controlling the SC transition from quiescence to activation involve different transcriptional/post-transcriptional, epigenetic, metabolic and proteostatic regulations, including autophagy [4]. Recent discoveries show that SC are a heterogeneous cell population, even within the same tissue, and that their cell fate depends on multiple intrinsic and extrinsic factors derived from the local and/or systemic environment [4]. Despite several mechanisms have been described, many other factors are emerging as relevant for SC self-renewal, activation, proliferation, and commitment in skeletal muscle during the regeneration process.

The Receptor for Activated C-Kinase 1 (RACK1) is a multifaceted member of the tryptophan-aspartate repeat family of scaffold proteins and shares significant homology to the β subunit of G-proteins [7, 8]. The functional role for RACK1 is to shuttle its binding partners to intracellular sites; however, another key aspect of RACK1 is the modulation of its partners, either promoting or suppressing the activity of bound enzymes. Although it has been initially isolated as a highly conserved intracellular adaptor protein for activated protein kinase C [9, 10], RACK1 was also found in ribosomes and in various sub-cellular structures, including the nucleus and midbody [11–15]. In ribosomes, RACK1 modulates the translation and controls de-novo polypeptide synthesis [8, 13, 16–18]. RACK1 has been also described to regulate key signals in multiple cellular functions including protein degradation, autophagy, proliferation, differentiation, survival, and development [7, 8, 16, 19]. In vascular smooth muscle cells, RACK1 acts on cell proliferation and contraction [20, 21] and is involved in hypertrophic responses in cardiomyocytes [22]. However, little is known about the role of RACK1 in functional and dysfunctional skeletal muscles and its actions during regenerative myogenesis remain under-investigated. An elegant genetic screen in ageing dystrophic muscles of the fruit fly *Drosophila melanogaster* identified RACK1 as one of dystroglycan and dystrophin interactors involved in cellular stress response [23]. RACK1 is also important in myoproteostasis, locomotor function, and longevity in Drosophila [24]. In vertebrates, a meta-analysis suggested a central role of multiple pathways, including RACK1, in the short-term atrophy network of skeletal muscle [25].

Muscle regeneration and rejuvenation therapies can benefit from greater knowledge about SC regulation. Using both mouse and *D. melanogaster* models, this latter as an ideal alternative framework to address questions that could not be easily approached with other organisms, we assessed here the expression/function/signaling of RACK1 in SC and its contribution to muscle regeneration after acute injury. Our result demonstrated for the first time that RACK1 is an important evolutionary conserved factor in adult SC differentiation both in vivo and in vitro. Indeed, RACK1 participates in skeletal muscle homeostasis by activating SC and is required for proper myogenesis of damaged skeletal muscle.

## Results

### Skeletal muscle growth and RACK1 levels

**Fig. 1:**
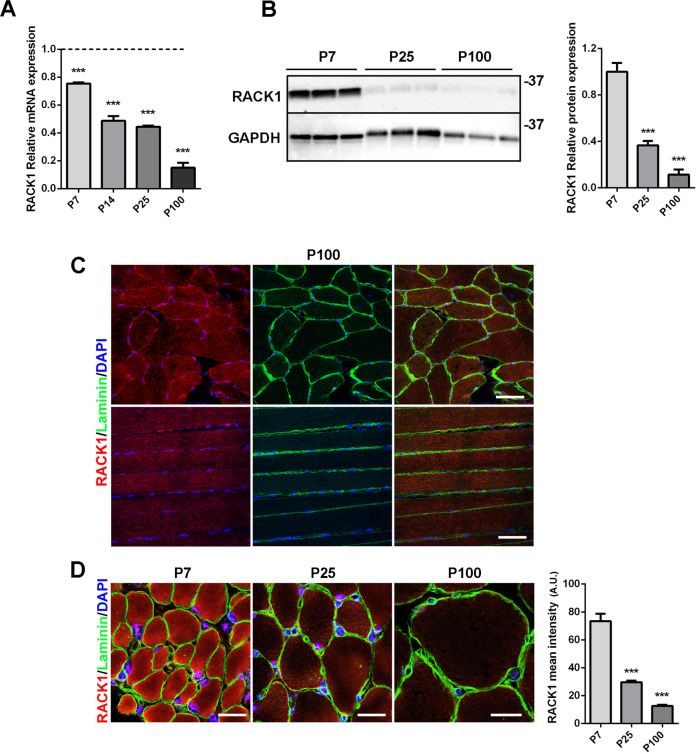
RACK1 expression in postnatal mouse muscle growth. **A** mRNA levels of RACK1 by RT-qPCR in TA muscle of P0, P7, P14, P25, and P100 mice. Results are expressed as fold change of P0 (dashed line). ***P < 0.001 vs P0 mice. **B** Western blot analysis of RACK1 in TA muscle of P7, P25, and P100 mice. GAPDH was used as internal standard. Right panel: densitometric quantification of RACK1; results are expressed as fold change of P7. ***P < 0.001 vs P7 mice. **C** Confocal fluorescence imaging of RACK1 (red), Laminin (green), and DAPI (blue) in transversal (upper panels) and longitudinal (lower panels) TA muscle sections of P100 mice (scale bars: 40 µm). **D** Confocal fluorescence imaging of RACK1 (red), Laminin (green), and DAPI (blue) in transversal TA muscle sections of P7, P25, and P100 mice (scale bars: 20 µm). Right panel: mean RACK1 intensity signals (A.U.: arbitrary units). ***P < 0.001 vs P7 mice. Images and quantitative data are representative of 6 ≤ *n* ≤ 10 mice.

In tibialis anterior (TA) muscles isolated from mice on post-natal days (P) 0–100, the mRNA of RACK1 was highest at P0 and the levels progressively decreased after birth, being very low at P25 and almost undetectable at P100 (Fig. 1A). In the same temporal window, RACK1 protein levels were significantly downregulated (Fig. 1B). Accordingly, transversal and longitudinal sections of TA muscle showed weak sarcoplasmic expression of RACK1 immunofluorescence in P100 mice (Fig. 1C). The post-natal muscle growth phase was characterized by an evident decrease of myofiber-associated RACK1 staining: during neonatal myogenesis (P7), in which muscle growth is sustained by SC proliferation and fusion, RACK1 is highly expressed while during adult myogenesis (after P21), when myofiber growth is less reliant on SC, RACK1 levels were significantly lower (Fig. 1D).

### RACK1 expression is up-regulated during SC activation

**Fig. 2:**
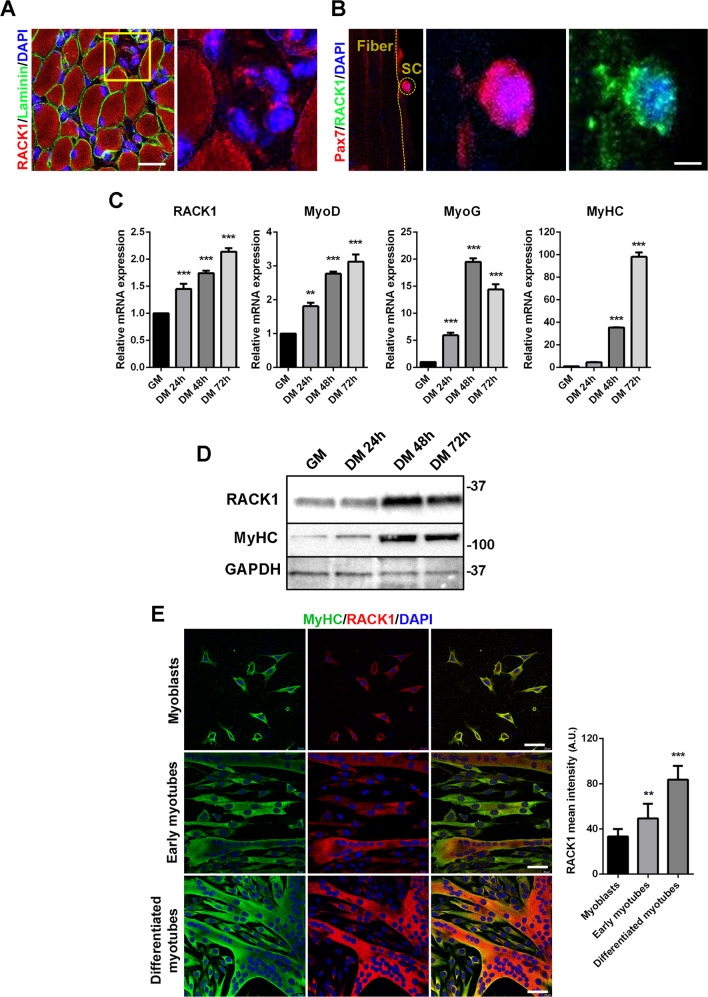
RACK1 expression in mouse SC and in deriving myotubes. **A** Confocal fluorescence imaging of RACK1 (red), Laminin (green), and DAPI (blue) in transversal TA muscle sections of P7 mice (scale bar: 20 µm). Insert represents enlarged image details of RACK1^+^ interstitial cells showed in the right panel. Images are representative of 6 mice. **B** Confocal fluorescence imaging of Pax7 (red), RACK1 (green), and DAPI (blue) in TA-isolated fiber with enlarged image details of double RACK1^+^ and Pax7^+^ SC showed in the right panels (scale bar: 40 µm). Images are representative of 20 ≤ *n* ≤ 30 experiments. **C** mRNA levels of RACK1, MyoD, MyoG, and MyHC by RT-qPCR in proliferating (GM) and differentiating (DM) SC at increasing times. Results are expressed as fold change of GM SC. **D** Western blot analysis of RACK1 and MyHC in GM and DM SC at increasing times. GAPDH was used as internal standard. **E** Confocal fluorescence imaging of RACK1 (red), MyHC (green), and DAPI (blue) in GM SC (myoblasts) and in early (24 h DM SC) and differentiated (48 h DM SC) myotubes (scale bars: 40 µm). Right panel: mean RACK1 intensity signals (A.U.: arbitrary units). **P < 0.01 and ***P < 0.001 vs GM SC. Images and quantitative data are representative of 8 ≤ *n* ≤ 10 experiments.

At P7, RACK1 staining in TA muscle was localized also in some interstitial cells, likely SC, that populate the growing tissue (Fig. 2A). In agreement with the striking connection between RACK1 expression and SC, we thus isolated single myofibers from TA muscle, since the myofiber culture system preserves the myofiber/stem cell association, which is an essential component of the muscle stem cell niche [26]. Immunofluorescence analysis clearly showed RACK1 staining on the periphery of myofibers in cells expressing Pax7^+^ (Fig. 2B), used as a marker for SC pool.

To dissect the expression of RACK1 during myogenesis, we examined primary SC obtained from mice at P25 and cultured in vitro with growth medium (GM). Differentiation of SC was then induced by a mitogen-poor differentiation medium (DM) [27] and monitored by phase microscopy for myotube formation (Supplementary Fig. S1A). As shown in Fig. 2C, transcript levels of RACK1 were detected in proliferating SC (GM) and progressively increased after 24–72 h of DM, concomitantly to MyoD, MyoG and myosin heavy chain (MyHC) myogenic markers [4, 5]. Similar results were obtained in western blot analysis showing that RACK1 protein peaked at 48 h of DM (Fig. 2D). Consistently, basal RACK1 levels in growing myoblasts significantly increased in MyHC-enriched multinucleated myotubes which are progressing towards mature differentiation, with the highest levels reached in fully differentiated myotubes (Fig. 2E).

### RACK1 expression changes in mouse skeletal muscle following injury

**Fig. 3:**
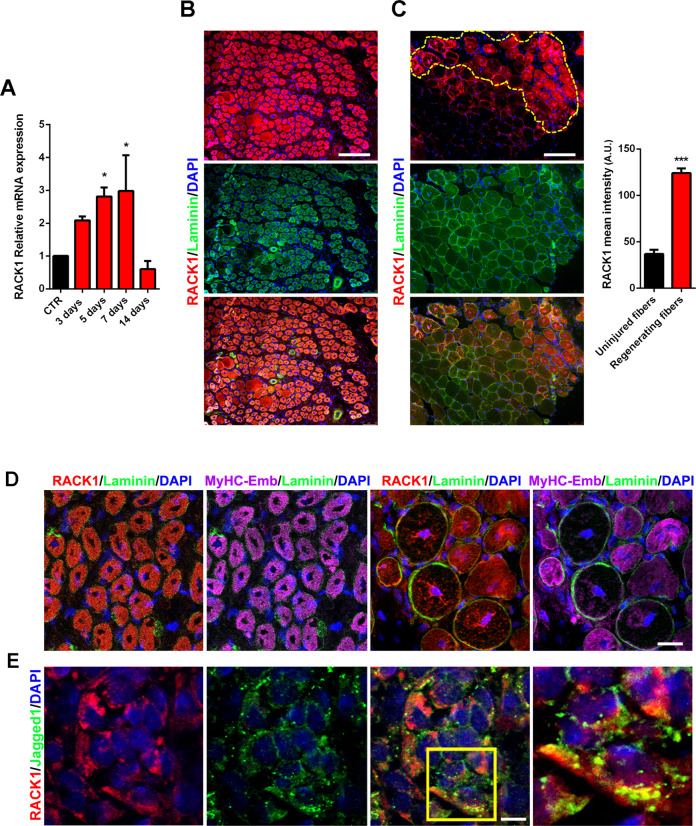
RACK1 expression in response to mouse muscle acute damage. **A** mRNA levels of RACK1 by RT-qPCR in TA muscle of CTX-injected mice at 3, 5, 7, and 14 days. Results are expressed as fold change of control (CTR, not injected) muscle (dashed line). Data are representative of 4 mice. *P < 0.05 vs CTR. **B**, **C** Confocal fluorescence imaging of RACK1 (red), Laminin (green), and DAPI (blue) in transversal TA muscle sections after 7 days post CTX injection (scale bars: 50 µm). The dashed yellow line represents the regenerating area. Right panel: mean RACK1 intensity signals in regenerating compared to uninjured fibers (A.U.: arbitrary units). ***P < 0.001 vs uninjured. **D** Confocal fluorescence imaging of RACK1 (red), Laminin (green), MyHC-Emb (purple), and DAPI (blue) in transversal TA muscle sections after 7 days post CTX injection (scale bar: 20 µm). **E** Confocal fluorescence imaging of RACK1 (red), Jagged1 (green), and DAPI (blue) of interstitial activated SC in transversal TA muscle sections at 7 days after CTX injection (scale bar: 5 µm). Insert represents enlarged image details of RACK1^+^/Jagged1^+^ cells showed in the right panel. Images and quantitative data are representative of 6 mice.

Accordingly to the modification of RACK1 during myogenesis in vitro, we verified this issue in vivo skeletal muscle. In particular, RACK1 expression in TA muscles of adult mice was monitored during regeneration induced by injection of cardiotoxin (CTX). As shown in Fig. 3A, acute damage led to a significant increase of RACK1 transcript during active regeneration at 5 and 7 days, while RACK1 content declined at 14 days, when the regeneration is almost complete [28]. Immunofluorescence analysis of muscle sections after 7 days CTX damage identified RACK1 accumulation in the centrally nucleated (regenerating) fibers and significantly higher levels of RACK1 were detected in regenerating fibers when compared with non-regenerating/uninjured fibers (Fig. 3B, C). Accordingly, RACK1 co-localized with the embryonal/developmental myosin heavy chain (MyHC-Emb), used as a proxy for regenerating fibers [29], and MyHC-Emb downregulation paralleled RACK1 expression (Fig. 3D). In addition, in the periphery of regenerating myofibers we also found interstitial cells showing co-localization of RACK1 and the Notch ligand Jagged1 (Fig. 3E), a marker for active SC [30, 31].

### Acute skeletal muscle injury and RACK1 expression in *D. melanogaster*

**Fig. 4:**
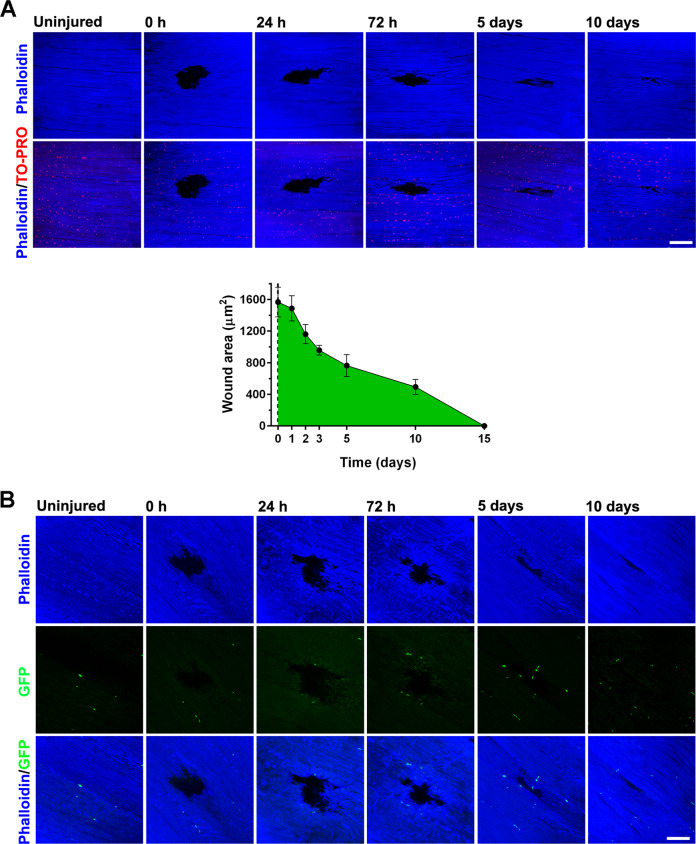
Acute skeletal muscle injury in *D. melanogaster*. **A** Confocal fluorescence imaging of Phalloidin (blue) and TO-PRO (red) in DLM longitudinal sections of Oregon wild-type fly strain before (uninjured) and after physical damage at increasing times. Low panel: time-course measurement of the wound area. **B** Confocal fluorescence imaging of Phalloidin (blue) and GFP (green) in DLM longitudinal sections of Zfh1>GFP flies before (uninjured) and after physical damage at increasing times. Scale bars: 20 µm. Images and quantitative data are representative of 30 ≤ *n* ≤ 60 flies.

Lineal descendants of muscle stem cells, equivalent of vertebrate muscle SC, are present in adult muscle of Drosophila as small, unfused cells observed at the surface and in close proximity to the mature muscle fibers [32, 33]. Based on our previous experience and complementary results simultaneously obtained in mouse and *D. melanogaster* models [34], localized stab injury of thorax muscles, i.e., dorsal longitudinal muscle (DLM), was carried out in young-adults (Oregon wild-type strain) using a small needle. Care was taken to restrict damage such that only few muscle fibers were affected and damage could regenerate. Time-course analysis of longitudinal DLM sections revealed that breaks of myofibers and disordered actin filaments were evident up to 3 days after injury although the physically induced wound was reduced in size (Fig. 4A). After 5 days, morphological regeneration was clearly active with the reconstitution of muscle fiber apparatus and the further repair of wound area. Only small remnants of the injury were visible at 10 days, when the actin filament arrangement almost completely recovered; while the damage was undetectable at day 15.

**Fig. 5:**
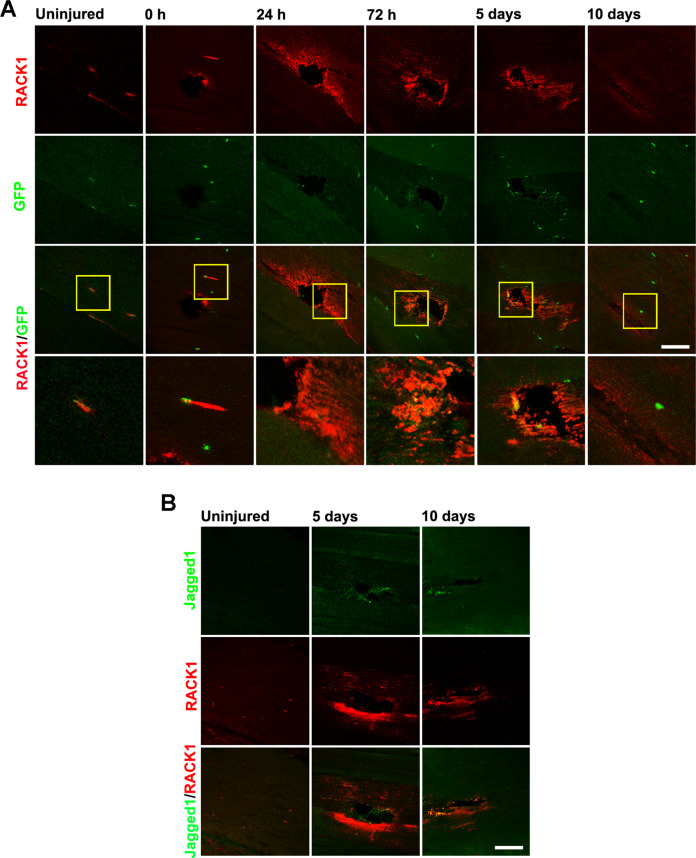
RACK1 expression in response to *D. melanogaster* muscle acute injury. **A** Confocal fluorescence imaging of RACK1 (red) and GFP (green) in DLM longitudinal sections of Zfh1>GFP flies before (uninjured) and after physical damage at increasing times. Inserts represent enlarged image details showed in the low panels. Images are representative of 30 ≤ *n* ≤ 40 flies. **B** Confocal fluorescence imaging of Jagged1 (green) and RACK1 (red) in DLM longitudinal sections before (uninjured) and after physical damage at increasing times. Scale bars: 20 µm. Images are representative of 15 ≤ *n* ≤ 20 flies.

To determine if muscle damage in Drosophila recapitulates the results obtained in mice, we repeated these experiments in Zfh1>GFP flies that allow to visualize zinc finger transcription factor (zfh1) patterns through a UAS-mCD8:GFP reporter. Zfh1 was shown to identify a population of muscle-associated cells in fly adult with progenitor-like properties [32, 35]. Uninjured DLM of Zfh1>GFP contained a small number of zfh1-expressing cells (GFP^+^, unfused SC) located peripherally in close proximity to the muscle fiber surface (Fig. 4B). In line with previous reports [32], during morphological regeneration after physical damage, some GFP-positive cells were progressively found around injured fibers indicating the recruitment of SC population. Immunofluorescence staining of RACK1 was faint in uninjured DLM of Zfh1>GFP flies although it somewhat co-localized with GFP^+^ SC (Fig. 5A). Noteworthy, a striking over-expression of RACK1 was associated with the injury, positioned around the damage and along the regenerating/differentiating fibers. Especially, RACK1 levels increased 1 day after damage and then progressively declined, appearing very low at 10 days when muscle tissue almost recovered. During the most active myogenesis program, i.e., 3–5 days, the presence of GFP^+^ cells high-expressing RACK1 was clearly detectable in the regeneration area around the wound. As shown in Fig. 5B, we found Jagged1 localized around the damaged area of DLM of Oregon flies during the active regeneration phase. Jagged1 protein is the ortholog of the Drosophila Notch ligand Serrate [30, 31] and it was used here to detect active SC in flies as in mouse muscle. Noteworthy, Jagged1 staining was detected in RACK1 overexpressing injured tissue.

### RACK1 depletion in SC impairs myogenic progression

**Fig. 6:**
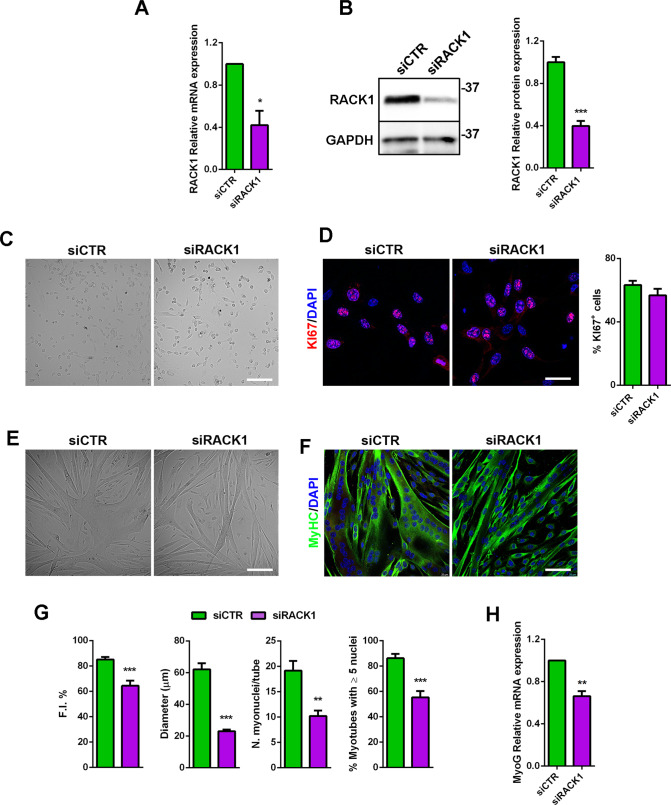
RACK1 downregulation affects mouse SC differentiation. Proliferating (GM) SC were transfected for 24 h with a RACK1-specific (siRACK1) or a non-targeting siRNA (siCTR). **A** mRNA levels of RACK1 by RT-qPCR. Results are expressed as fold change of siCTR. **B** Western blot analysis of RACK1. GAPDH was used as internal standard. Right panel: densitometric quantification of RACK1; results are expressed as fold change of siCTR. **C** Bright-field images (scale bar: 100 μm). **D** Confocal fluorescence imaging of KI67 (red) and DAPI (blue) (scale bar: 40 μm). Right panel: percentage of KI67^+^ cells on total DAPI-stained nuclei. SC were transfected for 24 h in GM with a RACK1-specific (siRACK1) or a non-targeting siRNA (siCTR), and then cultured for 48 h in differentiating (DM) conditions. **E** Bright-field images (scale bar: 100 μm). **F** Confocal fluorescence imaging of MyHC (green) and DAPI (blue) of forming myotubes (scale bar: 40 μm). **G** Fusion index (F.I.), mean myotubes diameter, mean number of myonuclei/myotube, and percentage of myotubes with 5 or more nuclei. **H** mRNA levels of MyoG by RT-qPCR. Results are expressed as fold change of siCTR. *P < 0.05, **P < 0.01, and ***P < 0.001* vs siCTR. Images and quantitative data are representative of 6 ≤ *n* ≤ 8 experiments.

To further understand the myogenic role of RACK1, we downregulated RACK1 in mouse SC by RNA interference. Hence, SC were transfected with either a RACK1-specific or a non-targeting siRNA and cultured in GM for 24 h. PCR and western blot analysis identified a significant decrease of RACK1 transcript (Fig. 6A) and protein (Fig. 6B) of about 60%. A similar downregulation of RACK1 was maintained after 24 and 48 h of DM (Supplementary Fig. S1B), indicating a persistent efficacy of siRNA effects during myogenic progression. As shown in Fig. 6C, D, RACK1 silencing did not seem to affect SC proliferation when compared to mock-transfected control and this is confirmed by unchanged KI-67 and cyclin D1 mRNA levels (Supplementary Fig. S1C). Subsequently, after siRNA transfection in GM, SC were switched to DM for 48 h to examine differentiation efficiency [27]. Both control and RACK1 siRNA SC formed multinucleated myotubes although we observed defective myotube growth in the presence of low levels of RACK1 (Fig. 6E, F). In particular, the fusion index, the myotube diameter, the mean number of nuclei/myotube, and the number of myotubes with 5 or more nuclei were significantly lower in RACK1 siRNA versus control SC (Fig. 6G). Consistently, the expression level of the myogenic marker MyoG was significantly reduced in RACK1 siRNA SC (Fig. 6H).

### RACK1 coupling to proteostasis operates on SC activation

**Fig. 7:**
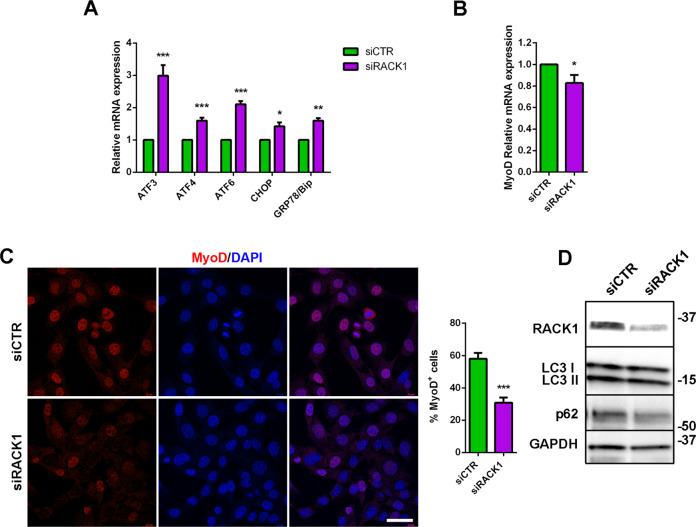
RACK1 downregulation in mouse SC is associated to UPR response. SC were transfected for 24 h in proliferating (GM) conditions with a RACK1-specific (siRACK1) or a non-targeting siRNA (siCTR), and then cultured for 24 h in differentiating (DM) conditions. **A**, **B** mRNA levels of UPR genes (ATF3, 4, 6, CHOP, and GRP78/Bip) and MyoD by RT-qPCR. Results are expressed as fold change of siCTR. **C** Confocal fluorescence imaging of MyoD (red) and DAPI (blue) (scale bar: 40 μm). Right panel: percentage of MyoD^+^ cells on total DAPI-stained nuclei. **D** Western blot analysis of RACK1, LC3I/II, and p62. GAPDH was used as internal standard. *P < 0.01, **P < 0.01, and ***P < 0.001 vs siCTR. Images and quantitative data are representative of 6 ≤ *n* ≤ 10 experiments.

To gain mechanistic insights, we tested whether RACK1 might regulate cellular stress response since it is a common pathway involved in SC function/dysfunctions, also interacting with catabolic systems such as autophagy [4]. After RACK1 siRNA transfection in GM, mouse SC were switched to DM for 24 h to examine different transcripts involved in muscle unfolded protein response (UPR), i.e., the activating transcription factors (ATF) 3, 4, or 6, the transcription factor C/EBP homologous protein (CHOP), and the ER chaperon GRP78/BiP [36]. As shown in Fig. 7A, RACK1 silencing significantly up-regulated the UPR markers when compared to control. To note, both the mRNA of MyoD (Fig. 7B) and its fluorescence immunostaining (Fig. 7C) were significantly reduced in SC after RACK1 silencing. In contrast, the sustained turnover of autophagy process in differentiating SC was not modified by low RACK1, since protein levels of the autophagy factors LC3I/II and p62 did not change (Fig. 7D).

### RACK1 is required for proper regeneration of *D. melanogaster* skeletal muscle following injury

The functional role of RACK1 was then investigated in vivo in Drosophila myogenesis upon physical damage. To this aim, UAS-RACK1 IR and Mef2-Gal4 flies were crossed as previously reported [24], producing F1 progeny in which the Rack IR construct became expressed following the Mef2 promoter, affecting late myogenesis, during the overt differentiation phase [37]. We thus obtained adult *D. melanogaster* phenocopies (Mef2>RACK1 IR) with silenced levels of RACK1 in muscle cells. As shown in Supplementary Fig. S2A, confocal microscopy did not reveal evident morphological changes in DLM structure. Accordingly, the climbing ability (vertical walking) of Mef2>RACK1 IR flies was not different from that observed in the presence of wild-type RACK1 (Supplementary Fig. S2B). Similar results were obtained when we compared the longevity of Drosophila strains (Supplementary Fig. S2C), indicating that alteration of RACK1 in adult skeletal muscle of flies does not impact on their general phenotype, locomotor ability, and lifespan.

**Fig. 8:**
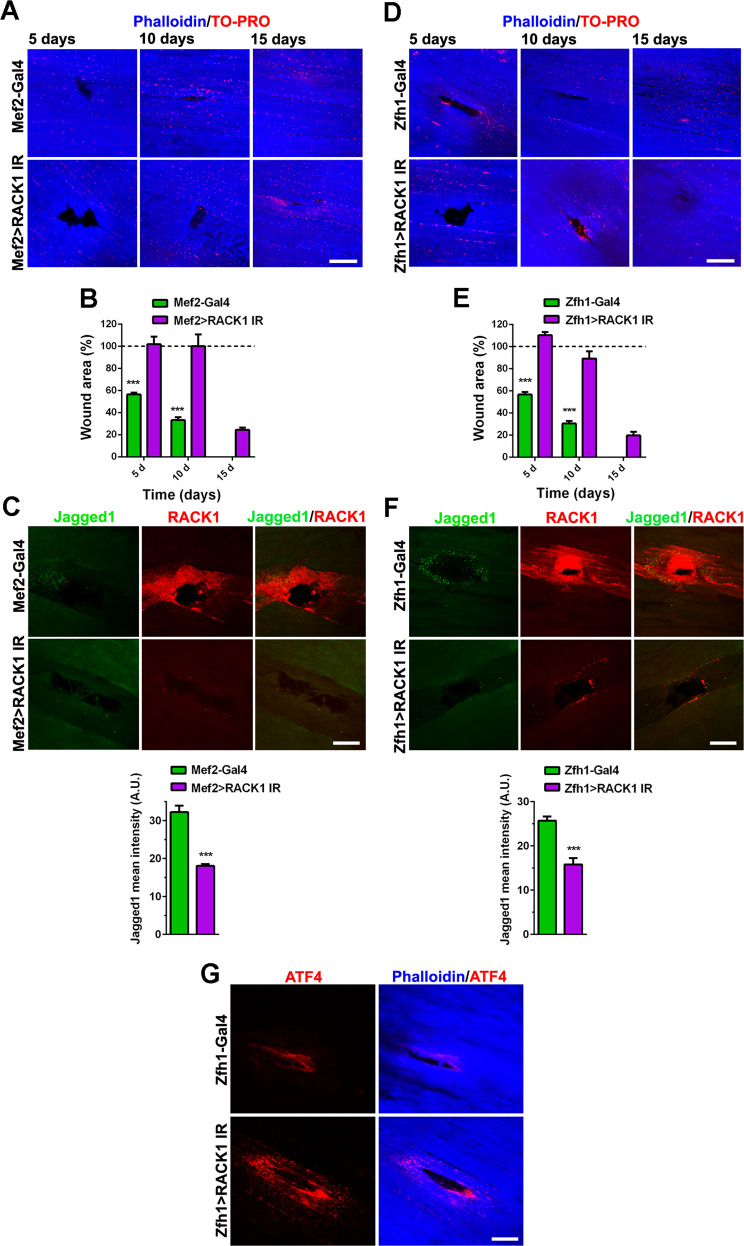
RACK1 downregulation affects *D. melanogaster* muscle regeneration. **A**, **D** Confocal fluorescence imaging of Phalloidin (blue) or TO-PRO (red) in DLM longitudinal sections of Mef2-Gal4/Zfh1-Gal4 (controls) and Mef2>RACK1 IR/Zfh1>RACK1 IR flies after physical damage at increasing times, and **B**, **E** time-course measurement of the wound area. Results are expressed as percentage of the wound area of the respective strain measured just after damage (*t* = 0 h, dotted line). ****P < 0.0001 vs Mef2>RACK1 IR/Zfh1>RACK1 IR. Images and quantitative data are representative of 20 ≤ *n* ≤ 30 flies. **C**, **F** Confocal fluorescence imaging of Jagged1 (green) and RACK1 (red) in DLM longitudinal sections of Mef2-Gal4/Zfh1-Gal4 (controls) and Mef2>RACK1 IR/Zfh1>RACK1 IR flies 5 days after physical damage. Lower panels: mean Jagged1 intensity signals (A.U.: arbitrary units) around the injury. ***P < 0.001 vs the respective control. Images are representative of 10 ≤ *n* ≤ 15 flies. **G** Confocal fluorescence imaging of ATF4 (red) and Phalloidin (blue) in DLM longitudinal sections of Zfh1-Gal4 (controls) and Zfh1>RACK1 IR flies 5 days after physical damage. Images are representative of 10 flies. Scale bars: 20 µm.

We then tested the muscle regeneration efficiency in RACK1 silenced flies. The injury wound in DLM of Mef2>RACK1 IR young adults was still clearly evident 5–10 days after injury and regenerated phenotype manifested at 15 days, while the onset-recovery of Mef2-Gal4 controls superimposed with the wild-type strains shown before (Fig. 8A). Accordingly, the repair process (size reduction) of the wound was significantly delayed in Mef2>RACK1 IR flies (Fig. 8B). As expected, at day 5 after injury, the immunofluorescence staining of RACK1 in the damaged area of DLM was clearly reduced in Mef2>RACK1 IR when compared with Mef2-Gal4 Drosophila (Fig. 8C). To note, Mef2>RACK1 IR injury exhibited faint expression of Jagged1 fluorescence, i.e., active SC. In particular, Jagged1 in the damaged area of Mef2>RACK1 IR Drosophila significantly decreased of about 44% versus control. These results suggest that RACK1 silencing impairs the activation of SC involved in the repair process.

To further assess whether the observed RACK1-interfered phenotypes were strictly associated to SC activity, we performed RNAi silencing of RACK1 specifically in SC crossing Zfh1-Gal4 and UAS-RACK1 IR lineage. Overall, the parameters observed with SC-specific downregulation of RACK1 in Zfh1>RACK1 IR Drosophila, i.e., the onset-recovery of DLM damage after physical injury (Fig. 8D, E), the immunostainings of RACK1/Jagged1, and the Jagged1 quantification at day 5 after injury (Fig. 8F), were superimposable with those seen in Mef2>RACK1 IR flies.

Finally, we took advantage of RACK1 silencing in SC of flies to test the involvement of ATF in RACK1-induced SC activation, since the UPR pathways are common between Drosophila and humans [38]. In particular, the Drosophila genome has a conserved ATF4 gene, also referred to as cryptocephal. As shown in Fig. 8G, the immunofluorescence staining of ATF4 in the damaged area of DLM at day 5 after injury was clearly overexpressed in Zfh1>RACK1 IR when compared with Zfh1-Gal4 Drosophila.

## Discussion

RACK1 has been previously associated to cell growth/proliferation and survival [7, 8, 16, 19]. For instance, neural development seems to require RACK1 [39, 40]. Thus, it is not surprising that RACK1 was expressed transiently in the skeletal muscle of post-natal mice, being abundant in the early phase of muscle growth and almost disappearing in adult mature fibers. When muscle develops SC proliferate and differentiate actively to form new fibers before entering a quiescent state. Pax7 is a pivotal regulator of SC specification [4–6]. The presence of RACK1 in interstitial Pax7^+^ SC tissue prompted us to hypothesize that RACK1 is important for skeletal muscle growth.

Once activated SC support growth and regeneration [3–6]. The dynamics of RACK1 levels in isolated adult SC of mice, progressively high during differentiation and low compared to proliferating conditions, suggested that RACK1 activity could be required for the myogenic program of SC, i.e., in nascent myotubes which are progressing towards mature differentiation. In adult skeletal muscle, the majority of SC are quiescent, but are poised for activation/differentiation in response to exercise, injury, or disease [3–6]. After muscle injury we found that RACK1 accumulated in mouse regenerating fibers during the remodeling phase, while it declined with the progression of regeneration. Consistent with these results, RACK1 accumulated in peripheral Jagged1^+^ cells, likely interstitial active SC that populate regenerating tissue. Indeed, Notch signaling, including Jagged1, is crucial in postnatal myogenesis for SC functions [30, 31]. Accordingly, Jagged1 expression was upregulated in regenerating fibers of mice after CTX-induced injury and during myoblast muscle differentiation in vitro [41]. Notwithstanding numerous differences in the growth of vertebrate and Drosophila muscles, there are remarkable similarities in the fundamental myogenic processes [42, 43]. For instance, SC are available for adult myogenesis in flies in response to damage, thus highlighting Drosophila as a model to understand muscle homeostasis [33, 43]. In addition, both the human and Drosophila genomes encode a single RACK1 gene and alignment of RACK1 protein sequence reveals strong conservation [44]. Thus, as alternative valuable in vivo tool, we set-up a Drosophila model with induced physical damage in skeletal muscles. As previously published in different fly strains [32], physical injury almost completely recovered after damage. Despite RACK1 being scarcely detected in intact or recovered fibers, it transiently increased in the wound area as a quick event during the repair process. RACK1 localized in the regenerating/differentiating fibers around the damage and in some zfh1-expressing SC. It was demonstrated that the activation of the normally quiescent zfh1 lineage anticipates the fusion with the damaged fibers in muscle repair, and that zfh1 was then down-regulated during the quiescent-active transition [32]. Our results were thus consistent with the role of RACK1 in the early phase, during which SC are committed to fuse and differentiate. Similar to mice, the possibility that active SC expressed high levels of RACK1 was confirmed by Jagged1 staining. Notch signaling is markedly conserved from Drosophila to humans, being the Drosophila protein Serrate the ortholog of the vertebrate protein Jagged1 [30, 31]. Following muscle injury in flies, SC undergo symmetric divisions through Notch signaling, that is also necessary for maintaining zfh1 expression [35]. Taken together these results indicated that RACK1 is a novel well conserved factor of SC whose expression mirrors SC activation and is closely associated with muscle growth/regeneration.

While inactivation of RACK1 was devoid of effects on adult SC proliferation, here we describe how RACK1-depleted SC were impaired in the differentiation process. Differentiation of SC occurs in 2 phases: single myoblasts fuse to form nascent myotubes, followed by recruitment of new nuclei to existing myotubes, leading to larger fully differentiated, multinucleated myotubes. The fusion index is a valid proxy of the first phase efficiency, while the mean number of nuclei/myotube and the percentage of myotubes with 5 or more nuclei are indexes of second phase effectiveness [27]. Our results in isolated mouse SC demonstrated that RACK1 promotes both the formation of myotubes and the accretion of nascent myotubes. Interestingly, in the presence of low RACK1, SC somewhat formed multinucleated myotubes although they cannot ultimate the differentiation program efficiently, as also suggested by the downregulation of MyoD and MyoG, early and late differentiation markers, respectively [4–6, 28]. Impaired differentiation ability of in vitro SC with defective RACK1 is consistent with the regenerative response obtained in vivo in Drosophila with depleted RACK1. Relevant to this study, homozygous knockout of RACK1 is lethal in mice [45]. In addition, the genetic handling of muscle cells may lead to death in rodents but not in Drosophila muscles [46, 47]. In both Mef2>RACK1 IR and Zfh1>RACK1 IR flies, in which RACK1 gene was silenced in all muscle cells (both progenitors and differentiated cells of the somatic muscle) or, specifically, in SC lineage, respectively, we observed a significant and similar delayed recovery of skeletal muscle after physical damage. Additionally, the low expression of RACK1 in the wound regenerative area significantly reduced Jagged1^+^ staining (active SC), further demonstrating the role of RACK1 in promoting SC activation and inducing efficient myogenesis.

The UPR is a conserved protein handling network that promotes the elimination of misfolded proteins and plays an important role in the survival, self-renewal, and differentiation of stem cells. It may regulate the SC transition to the activated state both in vitro and in vivo, also by interacting with catabolic systems such as autophagy [4, 36]. UPR and autophagy pathways play pivotal roles in regeneration of injured skeletal muscle [4, 36, 48]. While autophagy mechanisms in isolated mouse SC were not affected by RACK1 depletion, we observed increased levels of ATF3, 4, and 6 as well as CHOP and GRP78/BiP, thus indicating that RACK1-induced differentiation may involve, at least in part, the inhibition of UPR genes. In support of this hypothesis, we found that impaired levels of RACK1 in Drosophila SC induced ATF4 overexpression around the injury of regenerating muscles. As in other organisms, the stress response was shown to increase Drosophila ATF4 [49, 50]. To notice, our results suggest the evolutionary conservation of RACK1 and UPR coupling during SC activation. The increased levels of UPR signals may inhibit SC through repressing MyoD and in turn myogenesis [36, 51]. Accordingly, RACK1 silencing in isolated mouse SC inhibited MyoD. In multiple systems, RACK1 acts as a ribosomal scaffolding protein, modulating the translation of mRNAs and polypeptide synthesis, thus providing a hub integrating cell signaling and global protein [8, 13, 16, 17]. In this respect, RACK1 regulates myoproteostasis of Drosophila aging muscle, since it contributes to the modulation of misfolded protein aggregates, locomotor function, and longevity through the regulation of protein synthesis [24].

Collectively, in our study we found that RACK1 is mostly expressed in active SC and regenerating fibers respect to mature skeletal muscle cells, indicating that RACK1 is important for SC function. Specifically, we provide the first evidence that transient levels of RACK1 in SC are critical for efficient myogenesis to occur both in vitro and in vivo. Indeed, RACK1 favors the efficient progression of SC from a committed to a fully differentiated state, likely acting, at least in part, on UPR pathway. In this line, we demonstrated that RACK1 guarantees a proper SC-induced repair process in adult skeletal muscle after acute injury, while RACK1 defects determine delayed myogenesis and recover. Our findings highlight how the evolutionarily conserved RACK1 system deserves to be further investigated to achieve information translationally suited in muscle degenerative disorders and during aging.

## Materials and methods

### Chemicals

Phosphate buffer saline (PBS), Dulbecco’s Modified Eagle Medium (DMEM), penicillin-streptomycin, fetal bovine serum (FBS), and Horse serum (HS) were purchased from EuroClone (Pero, Italy). Chick embryo extract was obtained from United States Biological (Salem, MA, USA). Basic fibroblast growth factor (FGFb) was purchased from PeproTech (Cranbury, NJ, USA). The primary antibodies, including their suppliers, are listed in Supplementary Table S1. The primers pairs (Supplementary Table S2) were purchased from Eurofins (Vimodrone, Italy). The cocktail of protease and phosphatase inhibitors cOmplete and PhosSTOP was obtained from Roche Applied Science (Mannheim, Germany). Horseradish-peroxidase-conjugated secondary antibodies were purchased from Cell Signaling Technology (Danvers, MA, USA). Fluoroshield Mounting Medium containing DAPI and fluorescent phalloidin (#ab176752) were obtained from Abcam (Cambridge, UK). DAPI, Alexa-conjugates and TO-PRO™-3 Iodide (#T3605) were purchased from Invitrogen-ThermoFisher Scientific (Waltham, MA USA). Normal goat serum was obtained from Vector Laboratories (Newark, CA, USA). All other chemicals were from Sigma-Aldrich Merck (Darmstadt, Germany).

### Mice

C57BL/6 mice were purchased from Charles River Laboratories (Calco, Italy), housed in a regulated pathogen-free environment (23 ± 1 °C, 50 ± 5% humidity) with a 12 h light/dark cycle (lights on at 08.00 a.m.), and provided with food and water ad libitum. When indicated, both male and female mice were euthanized at different ages. Procedures were carried out in strict accordance with the Italian law on animal care (D.L. 26/2014, implementation of the 2010/63/UE) and approved by University of Milan Animal Welfare Body and by the Italian Minister of Health.

### *D. melanogaster* strains and husbandry

Wild-type (Oregon-R strain) and transgenic flies were obtained from Bloomington Drosophila Stock Center (BDSC, Indiana University Bloomington, IN, USA). As previously reported [52, 53], flies were routinely raised on a corn meal agar food (pH 5.5) at 25 °C, following standard mating procedures.

The fly strain Dmel\Mi{ET1}zfh1^MB07519^ (BDSC#25351; abbreviated as Zfh1-Gal4), which carries Gal4 insertion in the zfh1 locus, was crossed with UAS-mCD8::GFP flies (BDSC#5137) to obtain Zfh1>GFP progeny. In addition, flies expressing dsRNAi of RACK1 under UAS control (UAS-RACK1 IR; BDSC#38198) were crossed with flies expressing Gal4 under the control of the specific muscle driver DMef2 (Mef2-Gal4; BDSC#26882 and BDSC#27390) or with Zfh1-Gal4 flies. The F1 progeny obtained from these crosses (Mef2>RACK1 IR and Zfh1>RACK1 IR) was, at least in part, viable thus suggesting that the attenuation of RACK1 in muscles of flies does not induce drastic premature lethality.

### Primary mouse SC isolation and culture

As previously published [27, 54, 55], primary cultures of SC from 25 days old mice were obtained from dissociated muscles of hindlimbs and forelimbs by using the tissue dissociation protocol of gentleMACS™ Octo Dissociator with Heaters (Miltenyi Biotec, Bergisch Gladbach, Germany) followed by magnetic depletion of lineage ITGAM/CD11b (integrin alpha M), PECAM1/CD31 (platelet/endothelial cell adhesion molecule 1), PTPRC/CD45 (protein tyrosine phosphatase, receptor type, C) and LY6A/Sca-1 (lymphocyte antigen 6 complex, locus A) to exclude the Lin-negative population, using the Satellite Cell Isolation Kit (Miltenyi Biotec), according to the manufacturer’s protocols. SC were cultured in DMEM supplemented with 20% FBS, 3% chick embryo extract, 10 ng/mL FGFb and 1% penicillin-streptomycin on matrigel-coated plates at 37 °C with 5% CO_2_ for 4 days. To assess proliferation, SC were plated at a confluence of 1.5 × 10^4^ cell/cm^2^ in GM and cultured for 24 h. For the differentiation experiment, cells were plated at a confluence 5 × 10^4^ cell/cm^2^ in DM containing 2% HS instead of FBS and cultured for 24–72 h.

### RNA interference

According to the manufacturer’s protocol, RACK1 siRNA pool of 3 target-specific mouse Rack1 (Santa Cruz Biotechnology, Dallas, TX, USA) were mixed to Lipofectamine RNAiMax transfection reagent (Invitrogen-ThermoFisher Scientific). Control non-targeting siRNAs siRNA negative control (Santa Cruz Biotechnology) were also used. The mix was added to SC cells cultured in GM at a siRNA concentration of 10–50 nM for 24 h.

### Models of skeletal muscle injury

Acute muscle damage in mice was induced by injection of CTX from Naja pallida (50 μL, 10 μM) in TA muscle of 6–8 weeks old anesthetized mice, as previously described [56]. Mice were sacrificed 3, 5, 7, and 14 days after injury before muscle collection.

Fly muscle injury was performed as previously described [32, 57] with minor changes. Briefly, young-adults (2–3 days of adult age) flies were anesthetized with triethylamine and placed laterally under a stereo microscope. Only one of the two hemithorax was manually injured at DLM by inserting a thin pin (Minutien Pins-Stainless Steel/0.1 mm diameter #26002–10, Fine Science Tools, Heidelberg, Germany) for approximately 0.5 mm, avoiding extensive injury. Flies were recovered on standard food at 0–24–72 h and 5–10–15 days after injury before DLM collection.

### RNA extraction and RT-qPCR

The analysis of mRNA expression in TA muscle and SC of mouse was performed in PureZOL reagent (Bio-Rad, Hercules, CA, USA). Total RNA (500–800 µg) was retro-transcribed using iScript gDNA Clear cDNA Synthesis Kit (Bio-Rad). RT-qPCR was performed using SsoAdvanced™ Universal SYBR Green Supermix (Bio-Rad) and the CFX96 Touch Real-Time PCR Detection System (Bio-Rad). The primers pairs designed for RT-qPCR are detailed in Supplementary Table S2. Rpl38 and 36B4 have been used as housekeeping genes for normalization by using the 2^−ΔΔCT^ method.

### Western blot

TA muscles were homogenized with Ultra-Turrax (Ika Werke, Staufen, Germany) in a lysis buffer containing 20 mM Tris-HCl (pH 7.4), 10 mM EGTA, 150 mM NaCl, 1% Triton X-100, 10% glycerol, 1% Sodium Dodecyl Sulfate (SDS) supplemented with protease and phosphatase inhibitors. Protein extracts from cells were performed in RIPA buffer (10 mM Tris-Cl (pH 8.0), 140 mM NaCl, 1 mM EDTA, 0.5 mM EGTA, 1% Triton X-100, 0.1% sodium deoxycholate, 0.1% SDS) supplemented with protease and phosphatase inhibitors. Proteins were quantified using a BCA protein assay kit (Pierce, Rockford, IL, USA) following the standard protocol for western blotting. Thirty to fifty µg of total protein were loaded on 4–20 % polyacrylamide precast gels (Criterion TGX Stain-free precast gels; Bio-Rad). Proteins were transferred onto a nitrocellulose membrane using a Trans-Blot Turbo SystemTM (7 min at 2.5 A) and Transfer packTM (Bio-Rad). Primary antibodies used to probe membranes are indicated in Supplementary Table S1. After the incubation with the appropriate horseradish-peroxidase-conjugated secondary antibody [58], bands were visualized using the Clarity Western ECL substrate with a ChemiDoc MP imaging system (Bio-Rad). Bands were quantified for densitometry using the Bio-Rad Image Lab software. Uncropped western blots are provided in Supplementary Material.

### Fluorescence microscopy

For immunofluorescence experiments in TA muscle/single fiber and SC of mouse we followed a standard protocol [27, 59, 60]: samples were fixed with Paraformaldehyde (PFA) 4% for 10 min and permeabilized with 0.1% TritonX-100 in PBS 5 min, then blocked for 1 h in blocking buffer containing 5% normal goat serum and PBS. All primary antibodies (Supplementary Table S1) were diluted in blocking buffer and incubated overnight at 4 °C. Samples were washed three times with PBS and incubated with fluorophore-conjugate (Alexa-conjugates) secondary antibodies for 1 h at room temperature and nuclei were counterstained with DAPI (1:1000), for nuclei detection. Slides were mounted with Fluoreshield Mounting medium. Single myofibers were obtained from isolated TA muscles after 4% PFA fixation (1 h at room temperature): myofibers were dissected under a stereomicroscope and collected in PBS, then stained following the standard immunofluorescent protocol. Confocal images were acquired on a TCS SP8 System equipped with a DMi8 inverted microscope and a HC PL APO 40×/1.30 Oil CS2 (Leica Microsystems, Wetzlar, Germany) at a resolution of 1024 × 1024 pixels (single stack).

Drosophila thoraxes were collected and immersion-fixed for 2 h in cold 4% paraformaldehyde in 0.1 M Phosphate Buffer (PB) at 4 °C. Samples were then transferred to cold 20% sucrose in PB and stored at 4 °C for at least 24 h. Longitudinal sections of DLM (20 μm) were obtained by a cryostat, mounted onto positive charged slides, and stored at −20 °C until use. For immunostaining detection, sections were washed in PB and then pre-incubated for 1 h at room temperature with 5% BSA and 10% of normal goat serum in PB containing 0.5% Triton X-100. Pre-treated sections were incubated for 48 h at 4 °C with the primary antibodies listed in Supplementary Table S1 in PB containing 0.5% Triton X-100. GFP antibody was used to enhance the signal of fluorescent SC in Zfh1>GFP flies. Following washes in PB, the sections were incubated in the appropriate fluorophore-conjugate (Alexa-conjugates) secondary antibodies in PB overnight at room temperature. Images were acquired by a LSM 710 confocal microscope and a Plan-Apochromat 63×/1.40 Oil DIC M27 or EC Plan-Neofluar 40×/1.30 Oil DIC M27 (Carl Zeiss, Oberkochen, Germany) at a resolution of 1024 × 1024 pixels. The distance between adjacent focal planes (z-stacks) was set at 1 µm. Fluorescent phalloidin (F-actin staining, 1:1000) was used to observe Drosophila muscle structure and TO-PRO™-3 Iodide (1:1000) for nuclei detection.

Incubation in secondary antibodies alone was routinely performed as a negative control. When indicated, images has been analyzed by ImageJ software (National Institutes of Health, Bethesda, MD, USA).

### Climbing assay and survival of *D. melanogaster*

Geotaxis was assessed using a climbing assay (negative geotaxis reflex in opposition to the Earth’s gravity) as previously published with minor modifications [61]. Climbing performance was assessed at day 2–3 of adult age (just after eclosion). Survivorship was documented throughout the adult life of the flies ending at 35 days. The numbers of dead flies per vial was recorded every 7 days.

### Statistics

Generally, sample size calculation was conceptualized with 5% alpha error, 80% power and appropriate effect strength. Samples were only excluded from analyses due to technical problems, e.g., pipetting error, loss/spill of samples, or defects in materials/hardware. F-test was performed to evaluate the homogeneity of variance and Shapiro-Wilk test was used for evaluating data normality. The statistical significance of raw data between the groups (completely randomized) in each experiment was evaluated using unpaired Student’s *t*/Mann–Whitney tests (single comparisons) or one-way ANOVA followed by the Tukey post-test (multiple comparisons). A *p*-value ≤ 0.05 is considered statistically significant. Data belonging from different experiments (at least 4 biological replicates, *n*) were represented and averaged in the same graph. The GraphPad Prism software package (GraphPad Software, San Diego, CA, USA) was used. The results were expressed as means ± SEM of the indicated *n* values.

## Supplementary information

Supplementary Figure S1

Supplementary Figure S2

Supplementary Table S1

Supplementary Table S2

Uncropped Western blots

## Data Availability

The data analyzed during this study are included in this published article and the supplemental data files. Additional supporting data are available from the corresponding authors upon reasonable request.
