# Supplementary material for: RACK1 is evolutionary conserved in satellite stem cell activation and adult skeletal muscle regeneration
Source: Cell Death Discov. 2022 Nov 18;8:459. doi: 10.1038/s41420-022-01250-8 (PMC9672362; doi:10.1038/s41420-022-01250-8)
Supplement: Supplementary file 1 — Supplementary Figure S1 [file 41420_2022_1250_MOESM1_ESM.pdf]

Figure S1

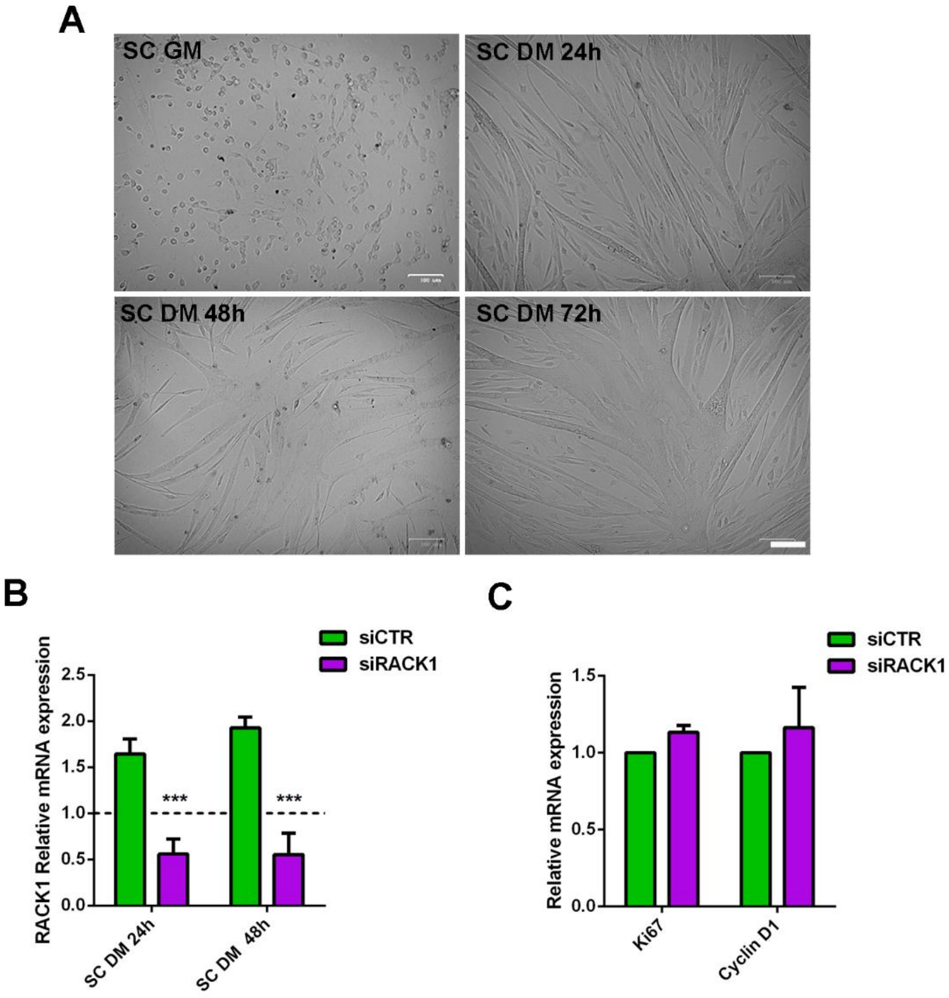

Supplementary Fig. S1. A Bright-field images of proliferating (GM) and differentiating (DM) mouse SC at increasing times (scale bar: 100 μm). **B** mRNA levels of RACK1 by RT-qPCR in mouse SC transfected for 24 h in proliferating (GM) conditions with a RACK1-specific (siRACK1) or a non-targeting siRNA (siCTR), and then cultured for 24 h or 48 h in differentiating (DM) conditions. Results are expressed as fold change of siCTR in GM SC (dashed line). ***P < 0.001 vs siCTR. **C** mRNA levels of RACK1 by RT-qPCR in GM mouse SC transfected for 24 h with siRACK1 or siCTR. Results are expressed as fold change of siCTR. Images and quantitative data are representative of 6 experiments.
