# Supplementary material for: RACK1 is evolutionary conserved in satellite stem cell activation and adult skeletal muscle regeneration
Source: Cell Death Discov. 2022 Nov 18;8:459. doi: 10.1038/s41420-022-01250-8 (PMC9672362; doi:10.1038/s41420-022-01250-8)
Supplement: Supplementary file 2 — Supplementary Figure S2 [file 41420_2022_1250_MOESM2_ESM.pdf]

Figure S2

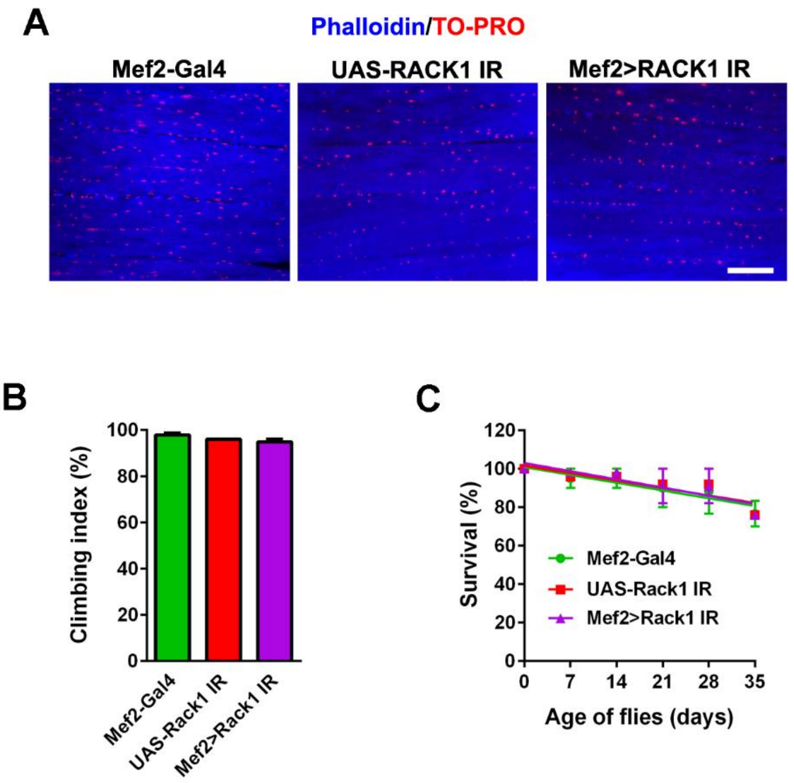

Supplementary Fig. S2. Analyses of Mef2-Gal4, UAS-RACK1 IR and Mef2>RACK1 IR young adult *D. melanogaster* strains. **A** Confocal fluorescence imaging of Phalloidin (blue) and TO-PRO (red) in DLM longitudinal sections (scale bar: 20 µm). Images are representative of 20≤n≤30 flies. **B** Climbing ability (vertical walking) expressed as the percentage of flies that climbed up to the 15 cm mark of the vial after 60 s. **C** Percentage viable animals throughout the adult life ending at 35 days. Data are representative of 40≤n≤50 flies.
