# Supplementary material for: RACK1 is evolutionary conserved in satellite stem cell activation and adult skeletal muscle regeneration
Source: Cell Death Discov. 2022 Nov 18;8:459. doi: 10.1038/s41420-022-01250-8 (PMC9672362; doi:10.1038/s41420-022-01250-8)
Supplement: Supplementary file 3 — Supplementary Table S1 [file 41420_2022_1250_MOESM3_ESM.pdf]

Supplementary Table S1. Primary antibody information.

Antibody	Host	Diluition*	Source	Cat. No
ATF4 (D4B8)	Rabbit	1:100 (IF d)	Cell Signaling (Danvers, MA, USA)	11815
GAPDH	Rabbit	1:5000 (WB m)	Santa Cruz Biotechnology (Dallas, TX, USA)	sc-25778
GFP (9F9.F9), DyLight ™488	Mouse	1:1000 (IF d)	Rockland Immunochemicals (Limerick, PA, USA)	200-341-215
Jagged1	Rabbit	1:100 (IF m)	Santa Cruz Biotechnology	sc-390177
Jagged1	Goat	1:50 (IF d)	Invitrogen-Thermo Fisher Scientific (Waltham, MA USA)	PA5-46970
KI67	Rabbit	1:200 (IF m)	Abcam (Cambridge, UK)	ab16667
Laminin	Rabbit	1:100 (IF m)	Sigma-Aldrich Merck (Darmstadt, Germany)	L9393
LC3	Rabbit	1:1000 (WB m)	Sigma-Aldrich Merck	L8918
MyHC (MF20)	Mouse	1:50 (IF m)<br>1:1000 (WB m)	Developmental Studies Hybridoma Bank (Iowa City, IA, USA)	
MyH3 (MyHC-Emb)	Mouse	1:100 (IF m)	Santa Cruz Biotechnology	sc-53091
MyoD	Mouse	1:100 (IF m)	Santa Cruz Biotechnology	sc-32758
p62/SQSTM1	Rabbit	1:1000 (WB m)	Sigma-Aldrich Merck	P0067
Pax7	Mouse	1:10 (IF m)	Developmental Studies Hybridoma Bank	
RACK1 (D59D5)	Rabbit	1:100 (IF m)<br>1:1000 (WB m)<br>1:100 (IF d)	Cell Signaling	5432

* IF: immunofluorescence; WB: western blot; m: mouse; d: Drosophila.
