# Supplementary material for: RACK1 is evolutionary conserved in satellite stem cell activation and adult skeletal muscle regeneration
Source: Cell Death Discov. 2022 Nov 18;8:459. doi: 10.1038/s41420-022-01250-8 (PMC9672362; doi:10.1038/s41420-022-01250-8)
Supplement: Supplementary file 4 — Supplementary Table S2 [file 41420_2022_1250_MOESM4_ESM.pdf]

Supplementary Table S2. Primer pairs designed for RT-qPCR analysis.

Name	Gene accession N°	Primer sequence*	Amplicon size
36B4	NM_007475	F: 5’-AGGATATGGGATTCGGTCTCTTC-3’<br>R: 5’-TCATCCTGCTTAAGTGAACAAACT-3’	143 bp
ATF3	NM_007498.3	F: 5’-GACCCCTGGAGATGTCAGTC-3’<br>R: 5’-TCTGACTCTTTCTGCAGGCA-3’’	148 bp
ATF4	NM_009716.3	F: 5’-AGCAAAACAAGACAGCAGCC-3’<br>R: 5’-ACTCTCTTCTTCCCCCTTGC-3’	193 bp
ATF6	NM_001081304.1	F: 5’-GAACTTCGAGGCTGGGTTCA-3’<br>R: 5’-TCCAGGGGAGGCGTAATACA-3’	204 bp
CHOP	NM_007837.4	F: 5’-ATATCTCATCCCCAGGAAACG-3’<br>R: 5’-TCTTCCTTGCTCTTCCTCCTC-3’	188 bp
cyclin D1	NM_001379248.1	F: 5’-AAATGCCAGAGGCGGATGAGAACA-3’<br>R: 5’-TGGAAAGAAAGTGCGTTGTGCGGT-3’	204 bp
GRP78/Bip	NM_001163434.1	F: 5’-TGTGGTACCCACCAAGAAGTC-3’<br>R: 5’-TTCAGCTGTCACTCGGAGAAT-3’	220 bp
MKI67	NM_026472.4	F: 5’-ACCGTGGAGTAGTTTATCTGGG-3’<br>R: 5’-TGTTTCCAGTCCGCTTACTTCT-3’	126 bp
MyHC (Myh2)	NM_001039545.2	F: 5’-GCGAAGAGTAAGGCTGTCCC-3’<br>R: 5’-GGCGCATGACCAAAGGTTTC-3’	76 bp
MyoD	NM_010866.2	F: 5’-CTGGCGCCGCTGCCTTCTAC-3’<br>R: 5’-GGCCGCTGTAATCCATCATGCCA-3’	129 bp
MyoG	NM_031189.2	F: 5’-GACCCTACAGACGCCCACAATC-3’<br>R: 5’-ACACCCAGCCTGACAGACAATC-3’	127 bp
RACK1	NM_008143.3	F: 5’-GGATCTCAATGAAGGCAAGC-3’<br>R: 5’-TTGCTGCTGGTGCTGATAAC-3’	183 bp
Rpl38	NM_001048057.2	F: 5’-GAAGGATGCCAAGTCTGTCAA-3’<br>R: 5’-GAGGGCTGGTTCATTTCAGA-3’	173 bp

* F: forward, R: reverse.
