# Supplementary figures and images for: RACK1 is evolutionary conserved in satellite stem cell activation and adult skeletal muscle regeneration
Source: Cell Death Discov. 2022 Nov 18;8:459. doi: 10.1038/s41420-022-01250-8 (PMC9672362; doi:10.1038/s41420-022-01250-8)

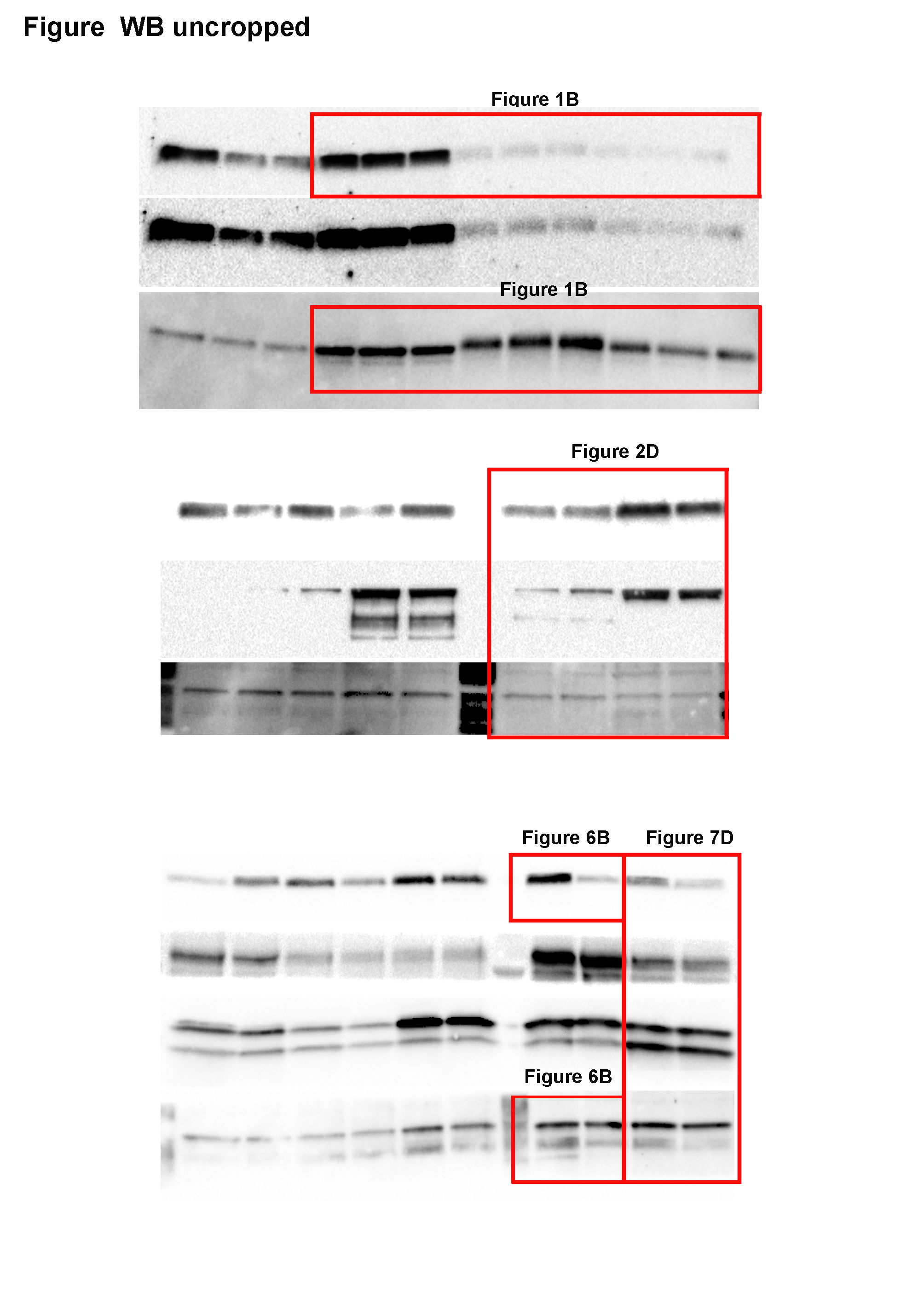

Supplement: Supplementary file 5 — Uncropped Western blots [file 41420_2022_1250_MOESM5_ESM.tif]
